# Repeatability and Reproducibility of a Double-Pass Optical Quality Analysis Device

**DOI:** 10.1371/journal.pone.0117587

**Published:** 2015-02-03

**Authors:** Chen-Chen Xu, Ting Xue, Qin-Mei Wang, Yi-Ni Zhou, Jin-Hai Huang, A-Yong Yu

**Affiliations:** The Eye Hospital of Wenzhou Medical University, Wenzhou, China; Sun Yat-sen University, CHINA

## Abstract

**Purpose:**

To evaluate the repeatability and reproducibility of a double-pass instrument (OQASII, Visiomereics SL, Spain), which objectively measures overall optical quality of the human eyes.

**Methods:**

The right eye of 119 healthy subjects with best corrected visual acuity of 20/25 or better was included in this prospective, comparative, observational study. Two separate tests with OQASII were conducted sequentially on the same day by two different examiners. A week later, the first examiner conducted the third measurement. All subjects underwent three consecutive tests during each session. The repeatability and reproducibility of the modulation transfer function cut off frequency (MTF cutoff), the Strehl ratio, the OQAS values (OVs) at contrasts of 100%, 20% and 9%, and the objective scatter index (OSI) were analyzed.

**Results:**

For MTF cutoff, Strehl ratio, OV100%, OV20%, OV9%, and OSI, the mean values were 39.32±9.75cpd, 0.22±0.06, 1.31±0.33, 1.33±0.39, 1.33±0.41, 0.60±0.42, respectively. Repeatability and reproducibility were good with a very low coefficient of variation and high interclass correlation coefficients (>0.88) for all parameters. Bland-Altman plots showed good correlation with 95% limits of agreement ranged from -6.04 to 6.78cpd, -0.05 to 0.05, -0.20 to 0.23, -0.29 to 0.32, -0.40 to 0.42, -0.23 to 0.21 in inter-observer, and -6.56 to 7.42cpd, -0.06 to 0.06, -0.22 to 0.24, -0.30 to 0.32, -0.35 to 0.34, -0.24 to 0.23 in inter-visit, respectively.

**Conclusion:**

The OQASII system yields excellent repeatability and good reproducibility for objective measurements of overall optical quality in clinic.

## Introduction

With the increasingly high demand of visual quality, many new instruments appeared to describe the optical quality such as point spread function, modulation transfer function (MTF), wavefront aberration, and ocular scattering. However, most of these instruments are not able to assess overall optical quality of the human eye only by an individual instrument. For example, aberrometors, the most frequently used instrument for optical quality assessment in ophthalmic practices, allow a measurement of aberrations rather than the changes of ocular scattering, resulting in overestimation of optical quality for the eye with significant scatter.[1] The Optical Quality Analysis System (OQAS, Visiomereics SL, Spain) provides parameters such as the MTF, Strehl ratio, and intraocular scattering based on the double-pass technique. It is the only available device that quantifies the combined effect of light scatter and optical aberrations, and objectively measures the overall optical quality of the human eyes.[1] OQASII is the latest version of OQAS with improvements in design, alignment, software and hardware, resulting in faster acquisition and more comprehensive analysis than the first generation of OQAS. The OQAS had been used to compare the results of optical quality between phakic intraocular lenses (IOLs) and LASIK, among different types of aphakic IOL.[2–4] Its measurement of intraocular scattering also had been used for cataract grading.[5, 6] Furthermore, it had been applied to patients with keratitis, dry eyes, as well as other pathologies.[7, 8] However, to our knowledge, no comprehensive data on the intra- and inter-session repeatability and reproducibility of OQASII has been published. In this study, the repeatability and reproducibility of the optical quality parameters provided by OQAS II were evaluated.

## Methods

All subjects enrolled in this prospective, comparative, observational study revealed no abnormal findings by a comprehensive ophthalmic screening examination. Enrolled subjects fulfilled the criteria, including age from 21 to 39 years-old, spherical equivalent (SE) from 0.00D to-9.50D and astigmatism less than 2.00D, stable refraction, the best corrected distance visual acuity (BCDVA) of 20/25 or better, pupil diameter larger than 4 mm in dim, without wearing rigid gas permeable contact lenses for at least 4 weeks or soft contact lenses for at least 2 weeks, no ocular disease, and no previous refractive surgery or systemic disease. Written informed consent was obtained from all subjects after explanation of the nature and possible consequences of the study. The research was approved by the Institutional Review Board of the Eye Hospital of Wenzhou Medical University.

### OQAS II Measurements

The OQASII measurements were performed in the right eye by two examiners. The first examiner measured subjects in the first week (session A), and the second examiner performed the measurements immediately after the first one (session B). A week later, the first examiner finished the third measurements (session C) in the same environment and the corresponding time with the first week. The examiners were blind to the results of each other and of each session. The subject’s eye was realigned at the beginning of each session. All subjects underwent three consecutive tests without realignment during each session. The subject was asked to open both eyes and fixate on a target. For each test, the software of the OQASII system captured six double-pass images to compute the final average optical quality.

### Optical Quality Parameters

The main outcome were the MTF cut off frequency (MTF cut off), the Strehl ratio, the OQAS values (OVs) at contrasts of 100%, 20%, and 9%, and the objective scatter index (OSI).

MTF is the ratio of contrast between the retinal image and the original scene. The MTF cut off provided by OQASII is the cut off frequency (cpd) at 1% of maximum MTF. In other words, it indicates the spatial frequency corresponding to the contrast of the retinal image at 1% of the original scene.[9]

The Strehl ratio is the ratio of central intensity of the point image between the measured eye and the ideal eye. The Strehl ratio provided by OQASII is the ratio of the area under the MTF curve between the measured eye and the ideal eye. A value of 1 indicates a perfect system influenced only by diffraction.[10]

The OV100%, OV20%, and OV9% represent the OQAS value calculated by the system at three contrasts commonly used in ophthalmic practice: 100%, 20% and 9%, respectively. The OV100% is the MTF cut off frequency divided by 30 cpd. The OV20% and OV9% are linked to 0.05 and 0.01 MTF values. Therefore, the three OVs are closely related to the MTF curve.

The OSI is the index quantified intraocular scattered light. The OSI provided by OQASII is the ratio of light intensity in the intensity curve between the annular area within 12 and 20 min arc and that within 1 min arc of the central peak.[6]

The higher the value of the MTF cut off, Strehl ratio and OVs, and the lower the OSI, the better the optical quality.

### Statistical Analysis

Statistical analysis was performed with commercial software (SPSS 18.0; SPSS, Chicago, IL, USA). The normality of data distribution was confirmed with the Kolmogorov-Smirnov test, and parametric statistical tests were used for data analysis. Descriptive statistics for continuous variables were calculated as means with standard deviations. The level of significance was P<0.05.

To assess intra-observer repeatability, the within-subject SD (Sw) of three consecutive measurements by the first examiner measured on the first day (session A) was calculated. Precision (repeatability coefficient) was defined as ±1.96 Sw. The within-subject coefficient of variation (CV, 100×Sw/overall mean) was also calculated. Further statistical analysis for the intrasession reliability was performed with intraclass correlation coefficients (ICC). The ICC was determined on the basis of analysis of variance for two-way mixed-effects model with an absolute agreement for consistency of individual measurements.

Differences between the session A and B (two different examiners during the same visit) were used to assess inter-observer reproducibility, and the differences between the session A and C (the same examiner during different visits) assessed inter-visit reproducibility. The Sw, precision, CV and ICC were calculated by the average of the means of each pair of readings.

The Bland-Altman method was used to assess inter-observer and inter-visit reproducibility of the system. The 95% limits of agreement (LoA) were defined as the mean difference ± 1.96 SD of the differences.

## Results

There were 119 subjects (119 right eyes), 59 male and 60 female, aged 26.8±3.9 years-old (range: 21 to 39 years-old). The mean SE of manifest refraction was-3.64±2.30D (range: 0 to-9.50D). All subjects had BCDVA of 20/25 or better. The mean value of the six optical quality parameters displayed in the Table 1.

**Table 1:** Intra-observer repeatability among three tests in each session for the parameters provided by OQASII.

	Mean value	Sw	Precision	CV(%)	ICC
MTF cutoff(cpd)	39.32±9.75	2.18	4.27	5.96	0.94
Strehl ratio	0.22±0.06	0.02	0.03	7.98	0.88
OV100%	1.31±0.33	0.07	0.14	5.94	0.94
OV20%	1.33±0.39	0.09	0.18	7.22	0.92
OV9%	1.33±0.41	0.10	0.20	8.02	0.90
OSI	0.60±0.42	0.05	0.09	9.49	0.98

### Repeatability

Table 1 presents the results of intra-observer repeatability. The ICCs for MTF cut off, OV100%, OV20%, OV9% and OSI were > 0.90. The ICC for Strehl ratio was close to 0.9. All CVs were less than 10, and all Sws and Precisions were within acceptable limit.

### Reproducibility

**Fig 1:**
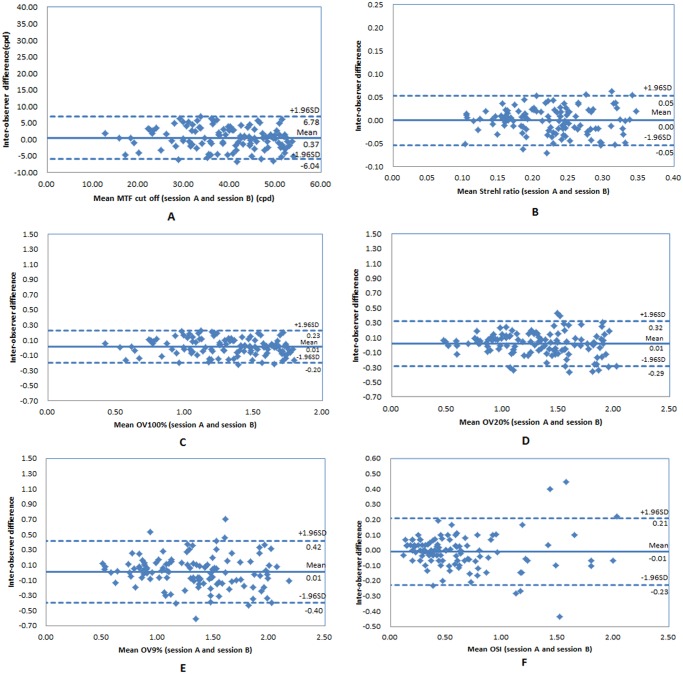
Bland-Altman plots of the mean differences between session A and B (inter-observer) were compared for parameters: MTF cut off (A), Strehl ratio (B), OV 100% (C), OV 20% (D), OV 9% (E), and OSI (F). The 95% limits of agreement are shown with dashed lines (ranged from-6.04 to 6.78cpd, -0.05 to 0.05, -0.20 to 0.23, -0.29 to 0.32, -0.40 to 0.42, -0.23 to 0.21, respectively), and the solid line represents the mean difference between these measurements.

**Fig 2:**
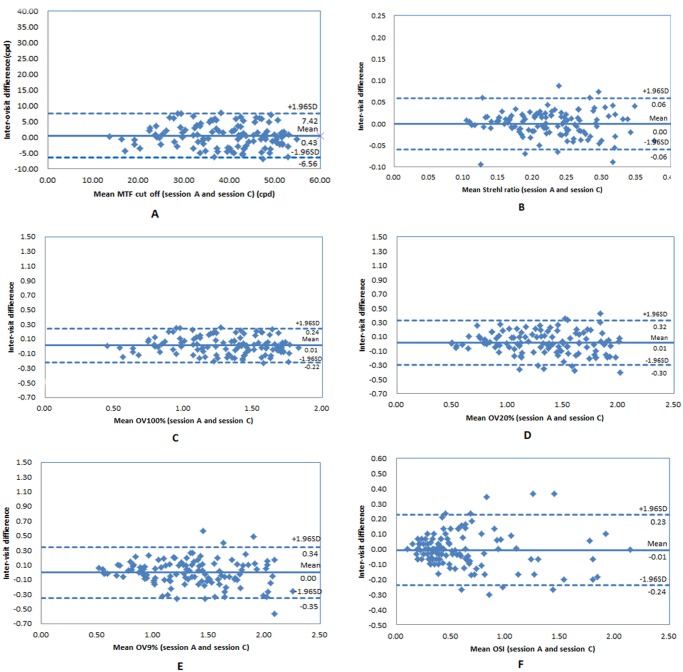
Bland-Altman plots of the mean differences between session A and C (inter-visit) were compared for parameters: MTF cut off (A), Strehl ratio (B), OV 100% (C), OV 20% (D), OV 9% (E) and OSI (F). The 95% limits of agreement are shown with dashed lines (ranged from-6.56 to 7.42cpd, -0.06 to 0.06, -0.22 to 0.24, -0.30 to 0.32, -0.35 to 0.34, -0.24 to 0.23, respectively), and the solid line represents the mean difference between these measurements.

Table 2 and Table 3 show the mean differences ± SD, Sw, Precision, CV, and ICC for the parameters provided by OQASII for inter-observer and inter-visit comparison, respectively. The inter-observer and inter-visit ICCs for OSI were >0.95, for MTF cutoff, OV100% and OV20% were >0.90, for Strehl ratio and OV9% were >0.88, respectively. The Bland-Altman plots (Figs. 1 and 2) illustrate the inter-observer and inter-visit variability were within acceptable limits in the clinical application.

**Table 2:** Results of inter-observer reproducibility of OQASII.

	Mean Difference	Sw	Precision	CV(%)	ICC
MTF cutoff(cpd)	0.37±3.27	1.90	3.73	5.33	0.95
Strehl ratio	0.00±0.03	0.02	0.03	6.98	0.90
OV100%	0.01±0.11	0.06	0.12	5.30	0.95
OV20%	0.01±0.16	0.09	0.17	6.52	0.93
OV9%	0.01±0.01	0.11	0.22	8.56	0.88
OSI	-0.01±0.11	0.05	0.10	9.55	0.97

**Table 3:** Results of inter-visit reproducibility of OQASII.

	Mean Difference	Sw	Precision	CV(%)	ICC
MTF cut off(cpd)	0.43±3.57	2.05	4.01	5.59	0.94
Strehl ratio	0.00±0.03	0.02	0.03	7.44	0.88
OV100%	0.01±0.12	0.07	0.13	5.50	0.94
OV20%	0.01±0.16	0.09	0.17	6.78	0.92
OV9%	0.00±0.19	0.10	0.19	7.09	0.91
OSI	-0.01±0.12	0.06	0.12	11.06	0.96

## Discussion

The use of validated and reliable measurement instruments is of critical importance for the clinical practice and interpretation of study results.[11] Therefore, it is crucial to evaluate and compare the repeatability and reproducibility of such devices, particularly before they become widely applied in clinical practice and research settings. With the increasing clinical application of OQASII, it is necessary to assess its repeatability and reproducibility on objective evaluation of optical quality.

Our results of optical quality are similar to Saad et al.[9] for the MTF cut off, Strehl ratio, OVs, and OSI, demonstrating that the healthy subjects have good optical quality. However, our results are slightly lower than that reported by Vilaseca et al.[12] The subjects were measured using the same series of trial lenses for correction of refractive error in this study. Trial lenses do not affect the repeatability, but may affect the accuracy of the results. In Vilaseca’s study, because the subjects wore spectacles for correction of spherical error between +0.25D to-0.50D, and cylindrical error no more than 0.50D, the low power trial lenses may lead to better optical quality.

### Repeatability

Our results revealed that OQASII had excellent repeatability in MTF cut off, OV100%, OV20%, OV9% and OSI, and good repeatability in Strehl ratio. Repeatability is the variability of the measurements obtained by one examiner while measuring the same item repeatedly. This is also known as the inherent precision of the device. The ICC, ranging between 0 and 1, is an overall index of repeatability. ICC is commonly interpreted as follows: ICC<0.4 indicates poor repeatability; 0.4<ICC<0.75 indicates fair to good repeatability; and ICC>0.75 indicates good to excellent repeatability. In clinic, ICC>0.90 means the device has excellent repeatability.[13] A value of 1 indicates perfect repeatability, meaning that each subject has the same value on test and retest. In this study, the ICCs were more than 0.90 for MTF cut off, OV100%, OV20%, OV9% and OSI, and close to 0.9 for Strehl ratio. The CVs of all parameters were low (less than 10), and the within-subject SD (Sw) were within acceptable limits in the clinical application.

The measurements of MTF cutoff and OV100% had high precision and low CV. OVs derive from MTF, so they directly relate to each other. Moreover, the higher the contrasts, the better the repeatability of OVs. In addition, the variability of OSI was larger than the Strehl ratio. This is similar with that reported by Vilaseca et al.[12] It might be explained by the difference in the computing method between OSI and Strehl ratio. The OSI is computed from the ratio between the light of peripheral annular region and the central peak of the double-pass image, but the Strehl ratio is calculated from the integration of the whole MTF profile divided by the area under the MTF curve of the aberration-free eye. For different sources, variations of the light in the double-pass image may have a great impact on the OSI. Meanwhile, the artificial pupil with a diameter of 4mm reduces the impact of pupil diameter on the MTF, decreasing the variability of the Srehl ratio.

### Reproducibility

Reproducibility is the variability of the measurement system caused by differences in examiner’s behavior. Mathematically, it is the variability of the average values obtained by several examiners (inter-observer) or one examiner in different times (inter-visit) while measuring the same item. In this study, the Bland-Altman plots showed the mean differences for all parameters closed to 0, and the 95%LoA were small for inter-observer and inter-visit. All CVs were low except OSI (but its ICC was the highest among all parameters). Sws were within acceptable limits in the clinical application. All ICCs were higher than 0.88. All of the results illustrate the OQASII system has good reproducibility.

The reproducibility was similar to repeatability, but inter-visit reproducibility was slightly worse than inter-observer reproducibility for MTF cut off, Strehl ratio, OV100%, OV20% and OSI. This may be explained by the microfluctuations of accommodation, small fixational eye movements, and instability of tear film.[14] The eyes need to be realigned in each session, so the eye position and accommodation may have small changes. The tear film is not the same in every session. These factors may influence the results. Moreover, the interval time for inter-observer (less than five minutes) is shorter than that for inter-visit (one week), so the above changes might be more significant for inter-observers comparison. In addition, methodologically, having the first examiner repeat the examination in one-week’s time may have an underlying bias.

In conclusion, the OQASII system yields excellent repeatability and reproducibility in MTF cut off, OV100%, OV20% and OSI, good repeatability and reproducibility in Strehl ratio and OV9%. It is a good objective measurement of overall optical quality in clinic. Further studies are required to analyze repeatability and reproducibility of this device for people with different age and different diseases.
